# Immunopathogenesis of Immune Checkpoint Inhibitor-Induced Myositis, Myocarditis and Myasthenia Gravis Overlap Syndrome: A Mechanistic Synthesis of a Mitochondrial Autoantigen Hypothesis

**DOI:** 10.7759/cureus.115848

**Published:** 2026-09-06

**Authors:** Umar Ismail

**Affiliations:** 1 Internal Medicine, Hywel Dda University Health Board, Haverfordwest, GBR

**Keywords:** cancer, ici, immune-related adverse event (irae), immunology, mitochondria damage, mmm syndrome, pembrolizumab-induced myasthenia gravis, proposed mechanistic pathways

## Abstract

Immune checkpoint inhibitors (ICIs) have improved outcomes for cancer patients. However, they can cause toxicities called immune-related adverse events (irAEs). One serious toxicity is the myocarditis, myositis and myasthenia gravis (MMM) overlap syndrome, an inflammatory syndrome involving the heart, skeletal muscles and neuromuscular junction. Most studies on the pathogenesis of myocarditis and myositis emphasise effector CD8+ T-cell driven inflammation while myasthenia gravis (MG) is believed to be a purely antibody-driven process. This approach has several challenges: (i) lack of a unified mechanism for a pattern of inflammation involving tissues with distinct immunologic and metabolic vulnerabilities; (ii) emphasis on CD8+ T-cell driven effector mechanisms without mechanistic exploration of inciting upstream events; and (iii) the high frequency of seronegative and fulminant MG phenotypes are not fully accounted for by the current models. To address these challenges, we propose a mitochondrial autoantigen hypothesis in which tumour- and therapy-associated cellular stress releases mitochondrial damage-associated molecular patterns (DAMPs) and proteins, promoting innate immune activation and antigen presentation. These mitochondrial proteins then act as shared autoantigens across the affected tissues once immune tolerance is lifted by ICIs. We believe these mitochondrial antigens activate a tissue-agnostic adaptive immune response through CD4+ T-cell-dependent mechanisms, which then depending on local tissue factors licence a B-cell response or a CD8+ T cell response as the dominant downstream effector mechanism. This framework is supported by evidence demonstrating the immunogenicity of mitochondrial proteins, and evidence that mitochondrial proteins can enter cellular antigen presentation pathways in both non-immune- and antigen-presenting cells. The hypothesis is further supported by evidence that a pre-existing circulating systemic pre-ICI immune state with expanded CD4+ effector-memory T cells and greater T cell receptor diversity predicts severe ICI toxicity. Additional conceptual precedent for this model is provided by analogous autoantigen generation processes seen in systemic lupus, anti-neutrophil cytoplasmic antibody associated vasculitis and antimitochondrial antibody-associated myopathy. The model draws on the established tissue vulnerabilities seen in mitochondrial disorders like mitochondrial encephalopathy lactic acidosis and stroke (MELAS) and electron transport chain disorders and uses this as a biological precedent for why mitochondrial perturbation may preferentially affect myocardium, skeletal muscle and other energetically demanding tissues. This hypothesis generates falsifiable predictions for biomarker discovery and provides a plausible mechanistic rationale for current treatment strategies targeting both humoral and effector T-cell suppression in MMM syndrome.

## Introduction and background

Immune checkpoints such as cytotoxic T-lymphocyte-associated antigen 4 (CTLA-4), programmed death receptor/ligand (PD-1/L1) and lymphocyte activating gene 3 (LAG3) play a central role in regulating immune response against virus-infected and transformed cells [1]. CTLA-4 inhibits CD28-mediated T-lymphocyte co-stimulation by competing for binding to CD80/86, inhibiting effector T-cell activation and proliferation [1]. PD-1, on the other hand, is an inhibitory receptor expressed by activated effector T-cells, regulatory T- cells (Tregs), B-cells, natural killer (NK) cells, dendritic cells (DCs) and non-immune tissues like the heart, liver, skeletal muscles and neuroglia [1]. Through interactions with its ligands (PD-L1 and L2), PD-1 regulates T-cell activation and is important in maintaining peripheral immune tolerance, a process exploited by cancer cells to evade immune detection [2]. LAG-3 is a ligand of major histocompatibility complex (MHC) class II molecules expressed on activated NK cells and T cells where it negatively regulates their activation. It also modulates effector T-cell function and Tregs suppressor activity [1]. LAG-3 antibodies thus augment CD8+ T cell function.

Immune checkpoint inhibitors (ICIs) targeting PD-1, PD-L1, LAG-3 and CTLA-4 have transformed cancer therapy by unleashing the immune system against cancer cells [1,2]. However, this robust immune response comes at a cost, on-target and off-target immune activation against normal tissues, leading to immune-related adverse event (irAEs). Although generally benign, these toxicities can rarely be life-threatening [3]. When they occur, these rare life-threatening toxicities are challenging to manage and, in some cases, lead to permanent ICI discontinuation. MMM overlap syndrome is one of the most lethal irAEs and represents a unique and aggressive entity characterised by synchronous inflammation involving myocardium, skeletal muscles and neuromuscular junction (NMJ) [4]. With an overall incidence of <1% among ICI-treated patients, its reported mortality is high at 40%-60% [4,5].

While mechanistic models exist to explain the individual components of MMM syndrome like myocarditis and myositis as stand-alone entities, to our knowledge, no prior work has attempted to synthesise a mechanistic explanation for the syndrome as a single immunopathological entity. The models for myocarditis and myositis to date have emphasised effector CD8+ T-cell activation, yet these frameworks do not fully explain what appears to be a highly specific pattern of cross-tissue inflammation affecting tissues with shared metabolic and immunologic vulnerabilities. While the CD8+ T-cell-centric model provides the mechanism of tissue damage, it does not fully explain the antigenic target(s) across these tissues or how the immune system gets sensitised to them. Current models also do not fully account for the high prevalence of antibody-negative, fulminant MG phenotypes commonly seen in MMM syndrome despite the belief that NMJ dysfunction is antibody-driven.

## Review

Methodology

To identify mechanistic clues scattered across multiple domains, we searched PubMed, Web of Science and Google Scholar from inception to August 2026 for literature on ICI-related MMM overlap syndrome. The search terms included combinations of 'immune checkpoint inhibitor,' 'myositis,' 'myocarditis,' 'myasthenia gravis,' 'overlap syndrome,' 'mitochondrial antigen,' 'ROS,' 'neoantigen,' 'reperfusion injury,' and 'immune tolerance'. Additional relevant articles were identified through manual citation chaining of key papers and review articles. Given the scarcity of mechanistic studies directly addressing MMM syndrome, we included a broad spectrum of evidence: systematic and narrative reviews, case series, case reports, translational immunology studies, preclinical experimental models, and mechanistic analogies from diseases such as antimitochondrial antibody myopathy, mitochondrial encephalopathy and stroke syndrome (MELAS), systemic lupus erythematosus (SLE) and antineutrophil cytoplasmic antibody (ANCA)-associated vasculitis. No formal quality appraisal was applied, as the aim was exploratory synthesis rather than systematic evidence grading. The findings were narratively synthesised around four themes: (i) T-cell recruitment and cytokine elaboration, (ii) antibody production, (iii) innate immune responses and complement activation and (iv) mitochondrial reactive oxygen species (ROS) and autoantigen generation as a unifying paradigm.

Current models of MMM overlap syndrome immunopathogenesis

Studies on ICI-related toxicities remain fragmented, largely focused on isolated, organ-specific mechanisms. To date, no single study has attempted to synthesise a unified disease mechanism for MMM syndrome. The most detailed studies are largely focused on the mechanism for myocarditis and myositis. The evidence from these studies suggests a multi-axis immune pathogenesis for the cross-tissue inflammation seen in myocarditis and myositis, which can be summarised in three sequential but overlapping steps.

First, effector T-cell recruitment and clonal expansion appears central: cases of fulminant ICI-associated myocarditis with concurrent myositis demonstrate a high frequency of infiltration with effector T-cells sharing T-cell receptors (TCR) directed against similar antigens in both tumour, myocardium and skeletal muscles, suggesting the existence of cross-reactive CD8+ T cells directed against antigens common to striated muscles [6,7]. However, the process by which these effector T cells come to be sensitised has not been explored in great depth. Complementary preclinical models implicate α-myosin-reactive cytotoxic T cells as sufficient to drive ICI-related myocarditis [8]. In their review, Rubio-Infante et al. suggested that tissue damage resulting from chemotherapy or radiotherapy prior to ICI administration may be the inciting event, resulting in tumour antigen exposure and epitope spreading [7]. ICIs then curtail the tolerogenic milieu, giving room for the autoreactive T cells to flourish. Evidence continues to accumulate, supporting the hypothesis that tissue damage during an immune response can lead to the priming of self-reactive T and/or B lymphocytes, regardless of the specificity of the initial insult [9].

Second, there is also accumulating evidence for B-cell activation and antibody production, particularly striational antibodies such as anti-titin, anti-Kv1.4 and anti-RyR, which are disproportionately enriched in MG patients who develop concomitant myositis or myocarditis [7,10]. However, a large percentage of patients with MMM syndrome test negative for these autoantibodies [4]. Muscle biopsy specimens from ICI-induced myopathies have also demonstrated complement deposition (C4d, C5b-9), and individual cases of MMM syndrome responsive to C5 blockade (eculizumab) further implicate an antibody-complement axis [7,11].

Third, cytokine elaboration and innate immune engagement (damage-associated molecular pattern (DAMP)-mediated neutrophil activation) appear to amplify the process: single-cell and multi-omics profiling of ICI-myocarditis has identified expanded populations of cytotoxic CD8+ T cells expressing granzyme/perforin alongside interferon-γ, tumour necrosis factor-alpha (TNF-α) and chemokines such as CXCL9/10, which recruit additional effector cells and perpetuate tissue damage [12-14].

Integrating these observations, a plausible summary is that ICI therapy unleashes cross-reactive cytotoxic T cells directed against shared muscle, cardiac and neuromuscular junction antigens, along with B-cell responses and complement fixation, while cytokine release and innate immune effectors amplify the inflammation.

Each of the mechanisms summarised in Table 1 is compelling within its own domain and provide important insights into effector pathways. Collectively, the studies reveal robust evidence for CD8⁺ T-cell and macrophage activity, cytokine release (interleukin 6 (IL-6), interferon gamma (IFN-γ), TNF-α), and humoral involvement across different manifestations of ICI toxicity.

**Table 1:** Summary of current mechanisms of ICI related cardiac and muscle toxicity syndromes NMJ: Neuromuscular junction; ICI: Immune checkpoint inhibitor; IL: interleukin; HLA: human leukocyte antigen; CCL: chemokine receptor ligand; TEMRA: terminally differentiated effector memory cells re-expressing CD45RA; MMM: myositis, myocarditis and myasthenia gravis syndrome; TCR: T-cell receptor; TNF: Tumor necrosis factor; MG: Myasthenia gravis; anti-AChR: anti-acetylcholine receptor; anti-HMGCR: anti-hydroxymethyl glutaryl-CoA receptor, IFN: interferon; PRISMA: Preferred Reporting Items for Systematic Reviews and Meta-analyses; IL: interleukin.

Reference	Study Type	Key Mechanistic Insights	Relevance to MMM Overlap
Sánchez-Camacho A et al., 2025 [15]	Narrative review	Described shared antigens between skeletal/cardiac muscle and NMJ that become targets in the context of ICI-induced immune dysregulation; increased CD8-to-CD4 T-cell ratios; autoantibody generation; complement activation; baseline cytokine abnormalities involving IL-17 and IL-6; genetic predisposition through certain HLA genes.	Provides a mechanistic bridge between cardiac and skeletal muscle injury via shared pro-inflammatory signaling axes.
Zhu H et al., 2022 [13]	Immunoprofiling (peripheral blood and tissue)	Expansion of cytotoxic CD8^+^ T effector cells re-expressing CD45RA (Temra CD8^+^ cells) in patients with ICI myocarditis. Elevated expression of proinflammatory chemokines (CCL5/CCL4) in the clonally expanded Temra CD8^+^ cells. Interactions of these cells with innate immune cells, including monocytes/macrophages, dendritic cells and neutrophils.	Defines immune signatures of myocarditis; supports potential blood-based biomarkers for myocarditis and by extension MMM syndrome.
Axelrod ML et al., 2022 [8]	Preclinical study + clinical correlation	Demonstrated CD8⁺ T cells with TCRs reactive to α-myosin; knockout and adoptive-transfer mouse models develop fatal myocarditis replicating ICI toxicity.	Confirms antigen-driven T-cell mechanisms potentially shared across myocardium and skeletal muscle
Lessomo FY et al., 2023 [16]	Narrative review	Summarised the current mechanistic hypothesis for ICI myocarditis, including shared antigens across tumour, skeletal and cardiac muscle recognised by T cells; findings of CD4⁺/CD8⁺ T-cell and macrophage infiltration with IL-6, IFN-γ, TNF-α upregulation in ICI myocarditis.	Reinforces mechanistic connections between myocarditis and myositis
Rubio-Infante N et al., 2022 [7]	Systematic review (not PRISMA-compliant)	Reviews autoantibody generation post-ICI and B-cell activation; implicates complement fixation.	Supports humoral immunity in MG and myositis; extends antibody-mediated injury concept to cardiac tissue.
Zheng J et al., 2025 [17]	Mechanistic intervention study	Highlighted the mechanisms of auto-reactive T-cell development and clonal expansion, and the role of specific cytokines like IFN-γ in macrophage polarisation. Explored the role of pro-inflammatory macrophage phenotypes and the chemokines they produce in the pathogenesis of ICI myocarditis. Discussed the role of inflammasomes in macrophage-driven inflammation.	The same mechanisms of autoimmunity targeting the myocardium can explain myositis.
Johnson DB et al., 2016 [6]	Case report (clinicopathologic)	Demonstrated the presence of T-cell/macrophage infiltrates in the myocardium of patients with ICI myocarditis. Immune infiltration was restricted to cardiac and skeletal muscle, suggesting shared antigen involvement. T-cell clones with TCRs reactive to tumour, myocardial and skeletal muscle antigens were demonstrated.	Findings raised the possibility that antigens (epitopes) present in the myocardium, skeletal muscle, and tumours were recognised by the same T-cell clones. Provided a mechanistic link between ICI myocarditis, myositis and anti-tumour inflammation.

However, none of these studies provide a unifying mechanistic framework that explains the synchronous multi-organ involvement observed in MMM syndrome. While T-cell infiltration, antibody deposition, and cytokine elaboration each contribute to tissue injury, these pathways do not explain why the same constellation of organs is repeatedly affected. There is also a lack of deep comparative analysis of these tissues beyond just inflammation. These tissues are embryologically and physiologically diverse; how and why does the immune system appear to mount a specific attack against them in the context of ICIs? This suggests potentially overlooked shared vulnerabilities.

A comparative analysis of the tissues consistently affected in MMM reveals convergent metabolic and immunologic features that may provide the missing mechanistic link (Table 2). If a common upstream trigger exists, it must align with shared biological properties across these tissues.

**Table 2:** Immunologic and metabolic comparison of tissues affected in MMM syndrome PDL-1: Programmed death receptor ligand 1; FasL: Fas ligand; TGF-B: transforming growth factor beta; APC: antigen-presenting cell;  ATP: adenosine triphosphate; MMM: myocarditis, myositis and myasthenia gravis; Treg: Regulatory T-cell.

Tissue/organ system	ICI-related toxicity profile	Oxidative metabolism/ mitochondrial dependence	Relative local immune tolerance	Dominant tolerance mechanisms	Local immune context (TRLs/innate presence)
Skeletal muscle	Uncommon but often severe (myositis)	High - oxidative. Fibres with dense mitochondrial content	Moderate-High	PD-L1 expressed by endothelium, Fas pathway, TGF-β	Sparse resident lymphocytes and APCs
Myocardium	Rare but highly fatal (myocarditis)	Very high - continuous aerobic metabolism	High	PD-L1⁺ cardiomyocytes, Fas pathway, complement regulation	Very low resident lymphocytes, few macrophages
Neuromuscular junction (NMJ)	Rare, often co-occurs with myositis/myocarditis (myasthenia gravis)	High, synaptic vesicle cycling and ion-pump ATP demand	High	PD-L1⁺ glial/peri synaptic cells, Fas pathway, complement inhibition	Minimal lymphocyte or innate immune presence

Mitochondrial autoantigen hypothesis

The convergent immunologic and metabolic characteristics summarised in Table 2 point towards a shared vulnerability across these tissues. Building on these shared characteristics, we propose a novel unifying mitochondrial autoantigen hypothesis to explain the synchronous inflammation seen in ICI-related MMM syndrome, built around a plausible common denominator, the mitochondrion: a metabolically central, immunologically active organelle enriched in all three tissues, with highly conserved protein signatures.

We hypothesise that mitochondrial immunogenic proteins, released and/or structurally modified during oxidative stress and tumour necrosis, may serve as the shared autoantigens at the centre of the inflammatory process seen in MMM syndrome. These antigens can drive a CD4+ effector T-cell response, which can drive both humoral immune activation and CD8+ T cell effector response across multiple organs in the context of immune checkpoint inhibition. This mitochondrial paradigm integrates existing mechanistic strands into a single explanatory framework for MMM syndrome. Candidate mitochondrial proteins are summarised in Table 3. 

**Table 3:** Candidate mitochondrial proteins/autoantigens and rationale for selection PDC-E2: Pyruvate dehydrogenase complex; OGDC-E2: oxoglutarate dehydrogenase complex; BCOADC: branched-chain alpha keto acid dehydrogenase complex; DLD: dihydrolipoamide dehydrogenase; ANT: adenine nucleotide transporter; TFAM: mitochondrial transcription factor A; AMA: anti-mitochondrial antibody; ROS: reactive oxygen species; NMJ: neuromuscular junction; mtDNA: mitochondrial DNA; DAMP: damage-associated molecular pattern; TLR9: Toll-like receptor 9; NLRP3: NLR family pyrin domain containing 3; VDAC1: voltage-dependent anion channel 1; ATP: adenosine triphosphate; AchR: acetylcholine receptor antibody; SDH-fp: succinate dehydrogenase flavo-protein subunit.

Candidate protein	Immunogenic rationale	Cross-tissue relevance
PDC-E2	Lipoylated; classical AMA target; highly immunogenic under ROS	Abundant in heart, muscle and synaptic cleft mitochondria
OGDC-E2/BCOADC-E2	Structurally homologous to PDC-E2; cross-reactivity well described	Muscle and heart metabolism
DLD (E3, dihydrolipoamide dehydrogenase)	Common E3 component across dehydrogenases; oxidation-sensitive	Heart, skeletal muscle enriched
ANT	Mitochondrial inner membrane protein (expressed by cardiomyocytes and skeletal muscle) in multiple isoforms). Strongly immunogenic	Demonstrated ability to induce myocarditis in EAM mouse models
TFAM (Mitochondrial transcription factor A)	mtDNA-binding protein; forms DAMP complexes activating TLR9/NLRP3	Bridges innate and adaptive immunity in all mitochondria-rich tissues
SDH-fp	Strongly immunogenic with documented autoantibody response in anti-M7 myocarditis and AchR-negative myasthenia gravis	Electron transport chain protein expressed by all MMM-affected tissues
ATP synthase (Complex V subunits ATP5A/B)	Reported surface expression; ROS-modifiable	Potential antibody target

Explaining the inciting event: the antigen trigger for ICI-induced MMM overlap may arise from tumour biology and from cytotoxic therapies

We hypothesise that the initial immune sensitisation to mitochondrial antigens may arise from the tumour microenvironment. Several mechanisms could facilitate this process. Ischaemic stress and systemic cancer therapies such as chemotherapy and radiotherapy can expose intracellular antigens to the immune system by inducing tissue injury and immunogenic cell death [18,19]. Solid tumours are metabolically active and may outgrow their blood supply, resulting in hypoxic stress and intermittent perfusion, which can generate ischaemia-reperfusion-like mitochondrial injury [18,19]. Similarly, ionising radiation and chemotherapeutic agents like anthracyclines can induce tumour cell death through the disruption of mitochondrial function among other mechanisms [20]. During periods of tissue stress, mitochondrial electron transport becomes impaired, while subsequent re-perfusion or re-oxygenation may promote a burst of mitochondrial reactive oxygen species (ROS) generation, including through reverse electron transport at complex I and through mechanisms amplified by succinate accumulation [21-24]. Opening of the mitochondrial permeability transition pore (mPTP) can further propagate ROS generation and mitochondrial dysfunction, contributing to sterile inflammation and cell death [25].

Beyond causing direct cellular injury, excessive ROS can oxidatively modify mitochondrial proteins and generate post-translationally modified (oxPTM) neoantigens, potentially creating novel epitopes that can elicit autoreactive immune responses [26-29]. In parallel, injured mitochondria also release damage-associated molecular patterns (DAMPs), including mitochondrial DNA (mtDNA), cardiolipin and N-formyl peptides, which can activate innate immune pathways involving TLR9 and the NLRP3 inflammasome, thereby amplifying local inflammation [30,31]. Mitochondrial proteins released or presented in an altered immunological context like the one described above could provide targets for cross-reactive adaptive immune responses since mitochondrial proteins are expressed by both tumour cells and normal tissues. This combination of antigen exposure, innate immune activation and tissue-specific vulnerability could provide a mechanistic basis for the organ tropism observed in MMM syndrome.

Consistent with this model, Johnson et al. reported CD8+ T-cell infiltration of the myocardium, skeletal muscle and tumour deposits in patients with ICI-associated myocarditis and myositis, with T-cell receptor (TCR) sequences suggesting recognition of shared antigenic epitopes in the affected tissues [6]. Complementing these findings at the systemic level, Lozano et al. found that elevated pretreatment abundance and TCR diversity of activated CD4+ effector memory T cells in circulation predicted severe irAE development irrespective of the affected organ system, across three independent melanoma cohorts [32]. While the study did not establish antigen specificity, the organ-agnostic nature of the association is consistent with shared, systemically distributed sensitising antigens such as the mitochondrial epitopes proposed here rather than independent, tissue-specific priming events. More recently, Booth et al. independently corroborated this pattern, finding that the elevated pretreatment PD-1+ T cells predicted subsequent irAE development in a separate melanoma cohort [33]. Together, these observations support a plausible framework in which tumour-associated antigen exposure initiates or amplifies an immune response that, following checkpoint blockade, can become systemically activated and target antigenically related tissues. The clinical correlation between ICI efficacy and some immune-related adverse events (irAEs) also lends weight to this paradigm [34,35].

Other risk factors involved in ICI-associated MMM syndrome must be acknowledged. Studies have implicated age, sex, ICI combination and certain human leukocyte antigen (HLA) haplotypes as potentially increasing the risk for MMM syndrome [4,6,36-38]. All affected patients in our institutional case series (n=4) were men [36]. In their systematic review, Pathak et al. reported a 67% male preponderance with a median age of 71 years [4]. Similarly, a recent systematic analysis of pooled patient-level data by Wei et al. reported a male preponderance of 70.6% and mean age 69.8±11.2 years at presentation (n=136) [37]. It is unclear whether this sex difference in incidence can be explained by differences in striated muscle and mitochondrial biology between men and women. Johnson et al. reported a 4.5 times higher risk of myocarditis with dual checkpoint therapy (Nivolumab/Ipilimumab) compared to single-agent use [6]. With respect to genetics, murine models of autoimmune myocarditis induced by defined mitochondrial epitopes (adenine nucleotide translocator 1, branched-chain α-ketoacid dehydrogenase kinase) are restricted to specific MHC class II haplotypes (H-2^a in A/J mice), with genetically similar but non-identical strains remaining resistant to identical immunisation protocols [38]. A human correlate exists in the association between HLA-DR4 and dilated cardiomyopathy independent of ICI exposure [39].

These observations suggest an interplay of factors that may best be explained by Rothman’s causal-pie framework, a model in which no single factor is independently sufficient to produce disease [40]. In this model, a minimal constellation of component causes must co-occur to complete a 'sufficient cause', and different constellations (different pies) may each independently produce the same clinical phenotype [40]. Within this framework, mitochondrial antigen exposure under checkpoint blockade is proposed as a necessary component present in essentially all ICI-treated patients experiencing tumour necrosis but insufficient by itself to produce MMM syndrome; clinical manifestation requires additional co-occurring components. We propose that male sex, advanced age, dual checkpoint blockade and permissive HLA class II haplotypes represent plausible completing components in this framework, individually insufficient but capable in combination of converting a latent mitochondrial antigen exposure into clinically manifest MMM syndrome.

Explaining the renal and hepatic exclusion

A simple review of our model immediately raises a question: Both the kidney and liver fit the profile proposed to explain the organ tropism seen in MMM syndrome, but why were they excluded? The brain and retina are also mitochondria-dense tissues, but why are they not part of the constellation? Although central nervous system and retinal immunotoxicities have been reported, these tissues are protected by anatomical and physiologic barriers completely different from those of MMM-affected tissues. We deliberately excluded the kidney from our model for two reasons. First, renal function is not systematically or consistently reported across MMM literature. The landmark systematic review of case reports by Pathak et al. noted that non-reporting of laboratory parameters could not reliably be distinguished from normal results [4]. Second, even when/if renal impairment is documented in MMM syndrome, attribution to a primary immunologic process would be confounded by the high prevalence of alternative, well-established causes of secondary renal injury in this population (pre-renal azotemia due to cardiogenic shock, nephropathy from rhabdomyolysis, sepsis-associated acute kidney injury (AKI) and nephrotoxic exposures in critical care). Similarly, the mechanism of identifying liver involvement relies heavily on raised transaminases. This is problematic for two reasons: (1) both aspartate transaminase (AST) and alanine transaminase (ALT) can be raised in myositis, making it difficult to distinguish true hepatic involvement and (2) when true hepatic dysfunction is established, its often difficult to separate complications of devastating organ failures seen in MMM syndrome, effects of hepatotoxic therapies utilised for immunosuppression, reactivation of pre-existing subclinical liver pathology and pure ICI-driven immune hepatitis, even with biopsy since there are no established pathognomonic features.

Immunologic precedents: lessons from systemic lupus erythematosus (SLE), ANCA-associated vasculitis and AMA-associated myopathy

To test whether the hypothesis aligns with known immunologic disease behaviour, we examined mechanistic precedents across three distinct immune-mediated diseases: SLE, ANCA-associated vasculitis and AMA-associated myopathy.

The well-characterised immunopathogenesis of SLE provides a proof of concept for our mitochondrial autoantigen hypothesis. SLE is a systemic autoimmune disease defined by the presence of autoantibodies directed against a wide array of intracellular nuclear and nucleolar components (e.g., dsDNA, Smith antigen, ribonucleoprotein (RNP)) [41]. The mechanisms by which these sequestered intracellular antigens become targets of a pathogenic humoral response provide a compelling proof of concept for how sequestered intra-organelle antigens can get unmasked and exposed to the immune system. Defective clearance of apoptotic/necrotic debris and neutrophil extracellular trap (NET) formation expose nuclear constituents (dsDNA, histones, RNPs), which engage endosomal TLR9 and other sensors, amplify type I interferon signalling, and promote autoantibody production [41]. Immune complexes are formed and deposited in target tissues, activate complement, with C4d and variably, C5b-9 reported in the affected organs [41]. This humoral-initiated, FcγR/complement-amplified inflammation with T-cell help broadly illustrates one component of our mitochondrial model, in which DAMPs (mtDNA, cardiolipin, N-formyl peptides) act as initial innate stimuli, and adaptive B and T cell response directed against co-released oxidatively modified mitochondrial proteins propagate tissue injury. This maps on to MMM syndrome because like nuclear antigens in SLE, mitochondrial antigens are normally intracellularly sequestered. Thus, SLE offers a conceptual template: Intracellular antigen exposure → innate sensing → antigen presentation/adaptive response → humoral/ effector T cell-mediated injury, applied here to mitochondrial rather than nuclear targets.

ANCA vasculitis supplies a micro-mechanistic precedent: oxidative stress and degranulation unmask sequestered intracellular enzymes (MPO, PR3), eliciting antibodies that drive FcγR-dependent effector- mediated injury [42]. We invoke ANCA in this case to illustrate how unmasking of sequestered antigens can initiate humoral-amplified tissue damage, an element that is supportive but not central, given our organelle focus. These precedents do not imply it is mechanistically identical to MMM syndrome; rather, they demonstrate plausibility that intracellular organelle antigens, once oxidatively modified and released, can become targets of adaptive and innate immunity. In the ICI context, PD-1/PD-L1 abrogation uniquely lowers the threshold for these events to culminate in CD4+ T cell-driven immune response directing a CD8+ dominant effector injury with humoral amplification.

Experimental precedents supporting mitochondrial autoantigens as drivers of multi-organ immune injury

While organ‐specific autoimmune diseases are believed to result from immune responses directed against self‐antigens specific to each organ, immune‐mediated responses targeting antigens expressed in multiple tissues provide a plausible mechanism for multisystem disease [38]. One such antigen is adenine nucleotide transporter (ANT), an inner mitochondrial protein heavily expressed in skeletal muscle, liver, myocardium, kidney and brain in four isoforms (ANT1-4) [38]. Basavalingappa et al. demonstrated that ANT1 can induce autoimmune myocarditis in A/J mice by generating autoreactive T cells. They showed that ANT1 encompasses multiple immunodominant epitopes (namely, ANT1 21-40, ANT1 31-50, ANT1 171-190 and ANT 1181-200). ANT1 21-40 in particular was found to be a major myocarditogenic epitope in immunised animals. The myocarditis-inducing ability of ANT1 21-40 was associated with the generation of T cells producing predominantly IL-17A, and the antigen-sensitised T cells could transfer the disease to naïve recipients [38]. These T-cells are also able to drive an antibody response. Indeed, production of ANT1 21-40 reactive antibodies increased by 12-20 fold (by day 21 to 52) in immunised mice compared to controls [38]. The authors also noted that the livers obtained from mice immunised with ANT1 21-40 showed multiple scattered lesions containing aggregates of lymphocytes as opposed to scanty inflammatory foci in the naïve animals (20.86±3.58 versus 2.10±0.69; P=0.0002) [38]. These findings are noteworthy because ANT1 antibodies have been detected separately in patients with dilated cardiomyopathy and are believed to impair myocardial calcium homeostasis by cross-reacting with myolemmal calcium channels [38]. Similarly, the same research group demonstrated that multiple peptide epitopes derived from mitochondrial branched chain α‐ketoacid dehydrogenase kinase (BCKDk) can become immune targets in the mediation of autoimmune myocarditis and/or hepatitis by generating autoreactive T cells in A/J mice in a separate paper [43]. Importantly, BCKDk- reactive immune complexes have all been detected in patients with dilated cardiomyopathy, providing preliminary human evidence that mitochondrial antigens can participate in cardiac autoimmunity [43].

Finally, AMA-positive inflammatory myopathy, in which antibodies target the E2 component of the pyruvate dehydrogenase complex (PDC-E2) provides a human precedent linking antimitochondrial autoimmunity with severe skeletal muscle and cardiac involvement [44]. Taken together, these experimental and clinical precedents establish that mitochondrial proteins can produce pathology across mitochondria-rich tissues. They therefore provide a mechanistic precedent for the mitochondrial autoantigen model proposed for ICI-induced MMM syndrome.

Cellular mechanisms exist for trafficking mitochondrial proteins to the cell surface for presentation on MHC proteins

Despite the presence of mitochondrial DNA, almost all mitochondrial proteins (99%), including ANT and several Table 3 candidates, are nuclear-encoded and are imported into mitochondria from the cytosol after translation [45]. Protein translocases in mitochondrial membranes are therefore essential for correct protein targeting and localisation [45]. The majority of precursor proteins (preproteins) are actively imported into mitochondria from the cytosol through the translocase of the outer membrane (TOM) complex, which forms a common entry gate for proteins that are subsequently targeted to various locations [45]. Under conditions of mitochondrial stress or impaired import capacity, these precursor proteins can accumulate in the cytosol as misfolded species, a phenomenon termed mitochondrial precursor over-accumulation stress [46], which renders them as substrates for the ubiquitin-proteasome system, the dominant route for generating peptides for MHC class I presentation [47]. This proteasome-dependent pathway is mechanistically independent of the MitAP/mitophagy-vesicle route. Together, these findings suggest that mitochondrial antigen presentation in MMM-relevant tissues may be reinforced by multiple, non-redundant cellular pathways rather than a single mechanism, increasing the plausibility that mitochondrial stress reliably generates MHC-I-presented peptides across affected organs.

Mechanistic work in other model systems has elucidated a dedicated mitochondrial antigen-presentation (MitAP) pathway, wherein mitochondrial-derived vesicles (MDVs) deliver organelle proteins to the endolysosomal system for MHC I loading [48]. Loss of the mitophagy regulators PINK1 and Parkin enhances this trafficking, leading to an accumulation of mitochondrial peptides on MHC I and activation of cytotoxic T cells [48]. In humans, PINK1 itself has been characterised as an autoantigen with defined HLA-restricted T-cell epitopes, confirming that mitochondrial proteins can be processed and recognised within the HLA context [48]. Recent studies have also shown that viral infections commonly associated with myocarditis and other autoimmune diseases, such as coxsackievirus B3 (CVB3) and severe acute respiratory syndrome coronavirus-2 (SARS-CoV-2), target mitochondria and are released from cells in mitochondrial vesicles that are able to activate the innate immune response [49]. These studies have shown that Toll-like receptor 4 (TLR4) and the inflammasome pathway are activated by mitochondrial components [49]. Evidence exists for autoantibodies against mitochondrial antigens in patients with myocarditis and dilated cardiomyopathy [49].

Crucially, several mechanisms could permit the trafficking of mitochondria-derived peptides onto MHC-II molecules on the cell surface of professional antigen-presenting cells (APCs) like macrophages and dendritic cells (DCs) [50]. This is a route that is potentially more parsimonious than and mechanistically independent of MDV-based trafficking. As discussed above, mitochondrial DAMPs (mtDNA, N-formyl peptides, cardiolipin) activate innate immune responses through pattern recognition receptors, including TLR9 (mtDNA), TLR2/TLR4 (formyl peptides, TFAM) and the NLRP3 inflammasome [51]. This activation upregulates MHC-II-dependent antigen presentation in both macrophages and DCs [52,53]. When these DAMPs are released alongside immunogenic mitochondrial proteins (including oxPTM species generated under the same conditions of metabolic and redox stress), the resulting innate activation promotes their phagocytic uptake, MHC-II loading and eventual presentation to CD4+ T cells [50,54]. The tumour microenvironment provides exactly this setting well before any checkpoint inhibitor is administered. Tumour-associated macrophages (TAMs) are chronically exposed to mitochondrial content released by dying and metabolically stressed tumor and stromal cells, and TAMs themselves constitutively express MHC-II and are established mediators of antigen presentation to CD4+ T cells within the tumour bed, with this presentation capable of reshaping both the CD4 response and the macrophage’s own phenotype in turn [55,56]. The tumour microenvironment therefore represents a plausible site of mitochondrial antigen acquisition by APCs and CD4+ T cell sensitisation: a chronic, DAMP-rich, MHC-II-permissive niche in which mitochondrial self-antigen exposure and antigen presentation are already ongoing [57]. Subsequent priming and expansion of antigen-specific CD4+ T cells may potentially occur in tumour-draining lymphoid structures.

We hypothesise that these activated CD4+ T cells in turn elaborate cytokines that drive both cellular (CD8+ T cell-dependent) and humoral (B cell-dependent) effector responses against tissues expressing the sensitising antigens. Under intact immune checkpoint function, this autoreactive T cell population is held in check by peripheral tolerance; checkpoint blockade lifts this restraint, allowing them to expand and propagate tissue injury. Supporting this sequence, Lozano et al. demonstrated that pretreatment CD4+ effector memory T cell abundance and T cell receptor (TCR) diversity predict severe irAE development in melanoma cohorts in an organ-agnostic manner [32]. This is direct clinical evidence that the immunologic groundwork for toxicity is laid well before treatment with ICIs begin. Taken together, these findings provide the mechanistic bridge linking mitochondrial stress, whether induced by reperfusion injury, cytotoxic therapy or metabolic overload, to adaptive immune activation. The result is a coordinated, multi-organ autoimmune process directed against mitochondria-associated epitopes, manifesting clinically as overlapping myositis, myocarditis and MG.

Precedents for organ tropism: inherited mitochondrial disorders and nucleoside reverse transcriptase inhibitor (NRTI) toxicities 

Inherited mitochondrial disorders such as MELAS and acquired mitochondrial toxicities induced by nucleoside reverse transcriptase inhibitors (NRTIs) provide clinical precedents for how mitochondrial dysfunction produces systemic, multi-organ disease. MELAS results from pathogenic mutations in mitochondrial DNA affecting oxidative phosphorylation, leading to stroke-like episodes, myopathy and cardiac dysfunction [58]. Hepatic involvement has also been reported [59]. These manifestations underscore the intrinsic multisystem nature of mitochondrial pathology, where metabolically active, mitochondria-rich tissues are preferentially affected.

NRTIs, including zidovudine and stavudine, can inhibit mitochondrial DNA polymerase γ, causing mtDNA depletion, impaired oxidative phosphorylation and energy failure [60]. The resulting toxicity localises to tissues with high oxidative metabolism - skeletal muscle and myocardium - closely mirroring the organ tropism seen in MMM. This pharmacologic model highlights that mitochondrial injury alone can replicate some of the tissue vulnerabilities even in the absence of autoimmunity.

It could be argued that autoimmunity against shared striational proteins is sufficient to explain this tissue overlap. While this may account for myocardial and skeletal muscle involvement, it does not sufficiently explain NMJ involvement. Mitochondria uniquely unify all three organs, aligning tissue vulnerability with metabolic activity and oxidative stress.

A mitochondrial antigen framework can account for the distinguishing features of seronegative MG in MMM syndrome

Idiopathic generalised MG is seropositive for AChR antibodies in the overwhelming majority of cases, with most “seronegative” cases subsequently found to harbour antibodies against muscle kinase (MuSK) or lipoprotein receptor related protein 4 (LRP4) [61]. In contrast, ICI-associated MG shows a high proportion of patients who are truly antibody-negative across all three established autoantigens despite unambiguous clinical and electrophysiological disease [4]. Additionally, the disease is disproportionately severe, often fulminant in presentation and frequently overlaps with myositis and myocarditis [4,37]. These observations are difficult to reconcile with a model in which antibody-mediated disruption of canonical NMJ targets is the main determinant of disease phenotype. A mitochondrial autoantigen framework offers a sound mechanistic explanation for this problem.

Direct clinical precedent exists for mitochondrial protein reactivity in MG. Gunji et al. found that antibodies against the flavoprotein subunit of mitochondrial succinate dehydrogenase (SDH-fp, the “64-kDa protein”) were present in 100% of generalised MG patients tested and 42% of ocular MG patients, with no correlation between SDH-fp reactivity and AChR antibody status in either group [62]. This provides evidence that humoral reactivity against a mitochondrial protein can accompany MG across both ocular and generalised phenotypes independent of AChR status. One possible interpretation consistent with our proposed model is that mitochondrial proteins, exposed and rendered immunogenic within a DAMP-rich inflammatory environment, could be taken up and presented by APCs on MHC-II to naïve CD4+ T cells, with the resulting CD4+ effector T cell population not committed to a single downstream effector modality. The resulting mitochondrial-antigen-specific CD4+ response could support both humoral and cellular effector arms in parallel. The relative dominance of either arm at the effector site is plausibly shaped by local factors including cytokine milieu, antigen-presenting cell subset and tissue-specific presentation route. Where this balance favours B cell help, the result is antibody-mediated disease of the kind captured by conventional AChR or antibodies against mitochondrial proteins such as SDH-fp. Where it favours CD8-dependent cytotoxic execution instead, muscle and NMJ injury could proceed via direct cytotoxic attack rather than antibody deposition, which can produce a clinically similar phenotype despite being poorly captured by conventional serologic testing.

This model offers a unified account of both distinguishing features of ICI-associated MG. Seronegativity becomes a predictable outcome of a shared upstream CD4+ T cell mechanism. The characteristic severity and multi-organ overlap (myositis, myocarditis) follow directly from the same upstream antigen being expressed across myocardium, skeletal muscle and NMJ, with a shared mitochondrial antigen-specific CD4+ T cell repertoire driving the coordinated immune responses across all three compartments, consistent with the clinical observation that ICI-myasthenia rarely occurs in isolation. This model generates a testable prediction: striated muscle and NMJ biopsies in seronegative ICI-MG should demonstrate CD8+ T cell infiltrates and evidence of mitochondrial antigen exposure at the site of injury, without a corresponding pattern of antibody deposition, a pattern that would not be expected if seronegativity in this population simply reflects assay insensitivity to an antibody that is, in fact, present.

**Figure 1:**
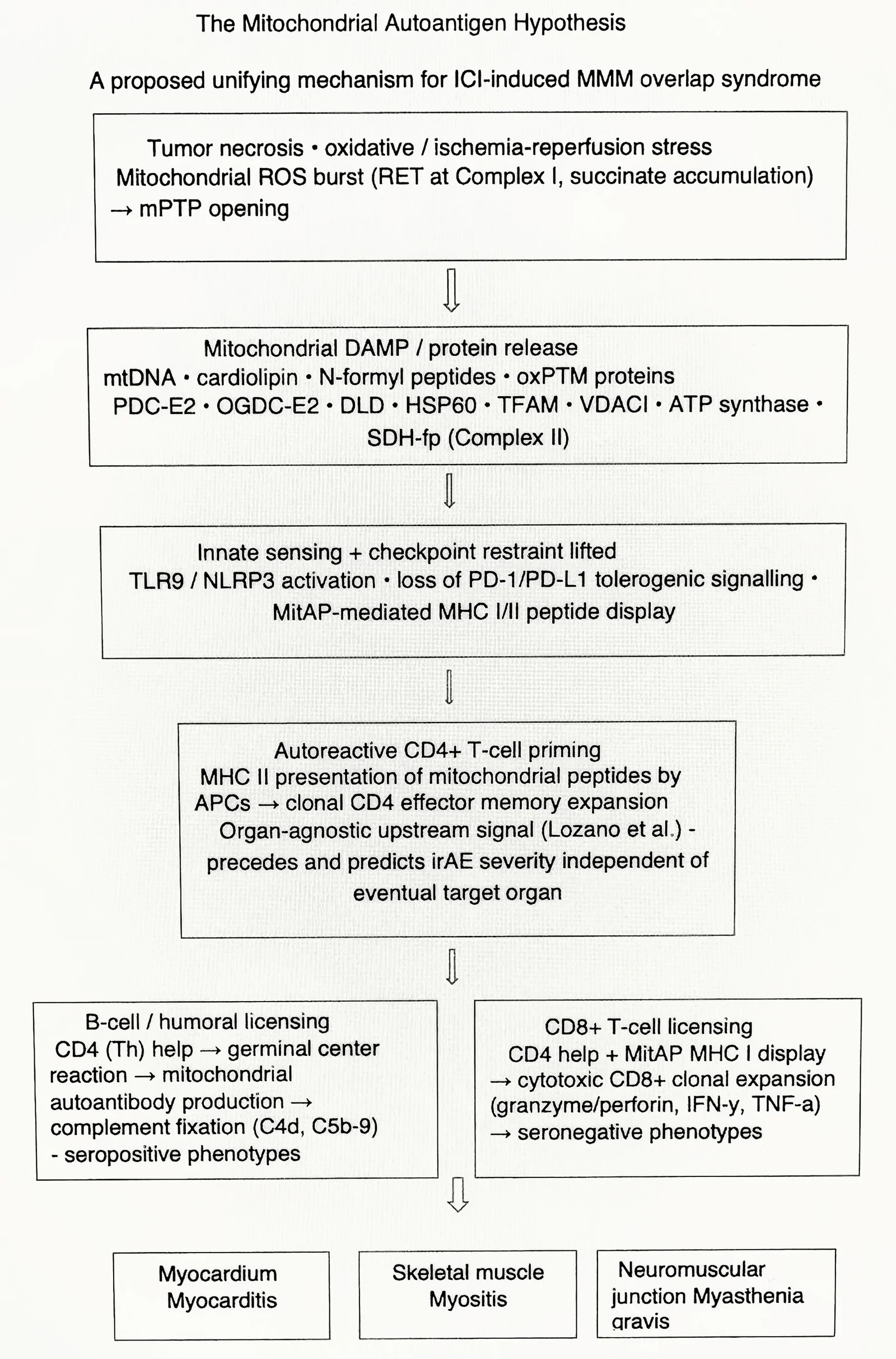
Visual schematic summarising our model ICI: Immune checkpoint inhibitor; MMM: myositis, myocarditis and myasthenia gravis syndrome; ROS: reactive oxygen species; RET: reversal of electron transport; mPTP: mitochondrial permeability transition pore; DAMP: damage-associated molecular pattern; mtDNA: mitochondrial DNA; oxPTM: oxidative post-translationally modified protein; PDC-E2: pyruvate dehydrogenase E2 subunit; OGDC-E2: oxoglutarate dehydrogenase E2 subunit; DLD: dihydrolipoid dehydrogenase; HSP60: heat shock protein 60; TFAM: mitochondrial transcription factor; VDAC1: voltage-dependent anion-selective channel protein 1; ATP: adenosine triphosphate; SDH-fp: succinate dehydrogenase flavoprotein subunit; TLR9: Toll like receptor 9; NLRP3: NLR family pyrin domain containing 3; PD1: programmed death 1; PDL-1: programmed death ligand 1; mitAP: mitochondrial antigen presentation; MHC: major histocompatibility complex; APC: antigen- presenting cell, irAE: immune-related adverse event; IFN: interferon; TNF: tumour necrosis factor.

Figure 1 summarises the complete mitochondrial autoantigen model and the proposed progression of events described throughout the manuscript.

Implications for therapy and diagnostics

There are currently no biomarkers that can reliably predict ICI response or risk of treatment-related toxicity. Our mitochondrial framework may have diagnostic and prognostic implications if proven. Circulating immunogenic mitochondrial proteins, along with sensitised CD4+ T cells, could serve as biomarkers to stratify the patient's risk of irAEs when detected. This requires tissue confirmation and investigation in prospective studies.

Current first-line therapy in MMM syndrome involves broad immunosuppression with high-dose IV steroids, which can be associated with substantial side effects and risk of opportunistic infections [3]. Additionally, the duration of immunosuppression may directly impact the response of the underlying tumour to ICIs [3]. Broadening our mechanistic understanding of this complex disease and the immune pathways involved allows better targeting of interventions to treat toxicities while potentially preserving anti-tumour efficacy. At present, intravenous immunoglobulin (IVIG), B-cell-directed therapies (e.g., rituximab), cytokine inhibitors (e.g. TNF and IL-6 inhibitors) and plasma exchange (PLEX) are employed as salvage therapies in steroid-refractory cases [3,4,63]. Our hypothesis, if proven, could provide a plausible rationale for considering some of these therapies earlier. Cytokine inhibitors could interfere with immune cell recruitment and activation, while IVIG could neutralise pathogenic Immunoglobulins and block Fcγ receptor engagement. PLEX could remove circulating autoantibodies, rituximab could suppress their production and complement inhibition (e.g., C5 blockade) could be explored as an adjunct in severe cases. Supporting this, C4d deposition in necrotic myocardium has been demonstrated in both clinical endomyocardial biopsy specimens and experimental models of ICI myocarditis, implicating classical complement pathway activation as a pathogenic mechanism [6,7]. These histopathologic observations suggest that antigen-antibody complex formation and complement fixation may directly contribute to cardiomyocyte injury, providing a rationale for therapeutic approaches targeting humoral immunity [4,7,64]. These suggestions are exploratory and should be taken with appropriate caution and will require validation in large prospective studies.

Closing perspective

Table 4 summarises the principal mechanistic models for MMM syndrome, each providing a significant insight into the disease process. Key limitations of each approach have been highlighted. Our mitochondrial framework provides granularity regarding the potential inciting event and how it unfolds. Other models highlighted in the table provide tissue-level evidence on the mechanism of injury downstream. 

**Table 4:** The principal mechanistic models currently used to explain MMM syndrome. NMJ: Neuromuscular junction; MG: myasthenia gravis; TCR: T-cell receptor; RyR: ryanodine receptor, AChR: anti-acetylcholine receptor antibody; MuSK: anti-muscle kinase; IVIG: intravenous immunoglobulin; PLEX: plasma exchange; MMM: myocarditis, myositis and myasthenia gravis syndrome; ICI: immune checkpoint inhibitor; IFN: interferon; TNF: tumour necrosis factor; IL: interleukin; scRNA cyTOF: time-of-flight cytometry; PD-1: programmed death 1; PDL-1: programmed death receptor ligand 1; TRM: tissue resident memory T-cell; CTLA-4: cytotoxic T-lymphocyte associated antigen 4; NEJM: *New England Journal of Medicine.*

Hypothesis	Core claim	Scope and limitations	Key predictions (falsifiable)	Tests / biomarkers	Therapeutic implications	Evidence notes
α-Myosin immunodominance/ shared muscle antigens	α-Myosin-reactive CD8⁺ T cells (±epitope spreading) drive myocarditis and secondarily myositis.	Explains strong cardiac-skeletal muscle coupling and T-cell clonality but not NMJ involvement or seronegative MG; complement positivity remains unaccounted for.	Dominant α-myosin-restricted TCRs; absence of NMJ antigenicity.	TCR sequencing; anti-striational antibody panels.	Favours T-cell–directed immunosuppression; limited role for humoral therapies unless antibodies present.	Supported in myocarditis/myositis; weak for NMJ disease.
Striational-antibody-centric model	Anti-titin/Kv1.4/RyR (±AChR/MuSK) antibodies mediate tissue injury.	Captures MG+myositis/myocarditis overlap and complement deposition but fails in seronegative and fulminant MG.	High striational/AChR/MuSK antibody frequency; complement deposition in the affected tissue.	Extended antibody panels; C4d/C5b-9 staining.	IVIG, PLEX, rituximab, or complement inhibition may benefit antibody-positive cases.	Subset phenomenon; cannot explain seronegative MG.
Bystander cytokine/myeloid amplification	ICI therapy causes IFN-γ/TNF/IL-6 overproduction and macrophage activation leading to bystander tissue damage.	Fits macrophage-rich histology and cytokine signatures but lacks antigen specificity or organ selectivity.	Cytokine elevation without antigen-specific TCR/antibody response.	Serum cytokine panels; scRNA-seq/CyTOF immune profiling.	IL-6R blockade or anti-cytokine therapy as adjuncts to steroids.	Amplifies injury but insufficient as a primary mechanism.
Immune-privilege failure (PD-1/PD-L1 axis)	Loss of PD-1/PD-L1 restraint unmasks latent autoreactive clones.	Explains temporal association with ICI use and multi-organ flares but not organ preference, seronegative MG, or complement deposition.	PD-L1 down-regulation across affected tissues without novel antigen discovery.	PD-L1; TRM activation markers.	Abatacept or CTLA-4 agonists may re-establish tolerance.	Provides a background permissive state rather than a direct trigger.
Tumour-self epitope overlap (neoantigen cross-reactivity)	Tumour antigens mimic self-antigens in affected tissues, driving cross-reactive CD8⁺ responses.	Explains TCR sharing between tumour and myocardium/muscle; cannot fully account for seronegative MG or complement activation.	Shared TCR clonotypes recognising tumor and self epitopes.	Comparative tumour-tissue TCR or immunopeptidome mapping.	Modulating tumor burden or T-cell activity may alter risk.	Supported by NEJM 2016 data showing tumor–muscle TCR overlap; incomplete unifying model.
Microvascular/endothelial injury	Endothelial PD-L1 loss triggers complement-mediated microvascular ischemia leading to secondary parenchymal damage.	Fits complement-positive, ischemic histology but not antigen-specific features or seronegative MG physiology.	Early endothelial activation and microthrombi preceding myocyte injury.	Endothelial activation markers; complement regulators.	Complement inhibition; anti-thrombotics (experimental).	Likely co-factor amplifying immune injury.

Limitations 

This review has several limitations that must be acknowledged. First, it is a hypothesis-driven narrative synthesis and therefore lacks structured search, inclusion/exclusion criteria and dual independent screening that would minimise selection bias. While a broad search of the literature was conducted, the possibility remains that some relevant studies may have been missed. Second, the evidence base informing this hypothesis is heterogeneous and fragmented, consisting of systematic and narrative reviews, case series, case reports, animal models and mechanistic analogies drawn from related fields, which makes it difficult to establish causality with confidence.

Third, there is currently no direct biomarker or experimental evidence confirming the role of mitochondrial autoantigens in ICI-induced MMM syndrome, and our proposed model should therefore be regarded as hypothesis-generating. Finally, the synthesis is limited by the absence of multi-omics or immunopeptidomics data that could validate mitochondrial protein presence in affected tissues. These limitations highlight the need for future translational and experimental work to substantiate, refine, or refute this proposed paradigm.

## Conclusions

MMM syndrome remains one of the most rare and lethal irAEs associated with ICIs. The pathophysiologic and immunologic processes underpinning this devastating syndrome are extremely complex. While current mechanistic models have advanced our understanding of these diseases, significant gaps still exist in our understanding of the initiating events. A detailed and nuanced understanding of these processes may generate opportunities for risk stratification and better targeting irAE treatment, which hopefully will preserve overall tumour response. Our mitochondrial autoantigen framework parsimoniously links organ tropism, innate immune activation and adaptive CD4+ T cell-dependent effector mechanisms. The model also proposes testable biomarkers and provides plausible mechanistic rationale for current therapeutic approaches. Validating or falsifying this hypothesis will require extensive human tissue studies.
